# G-Quadruplexes Formation by the *C9orf72* Nucleotide Repeat Expansion d(GGGGCC)_n_ and Conformation Regulation by Fangchinoline

**DOI:** 10.3390/molecules28124671

**Published:** 2023-06-09

**Authors:** Yun Zhang, Junliu Huang, Kainan Yu, Xiaojie Cui

**Affiliations:** 1 College of Life and Environmental Sciences, Minzu University of China, Beijing 100081, China; 2 Key Laboratory of Ecology and Environment in Minority Areas (Minzu University of China), National Ethnic Affairs Commission, Beijing 100081, China

**Keywords:** G-quadruplex, *C9orf72* hexanucleotide repeat, Fangchinoline

## Abstract

The G-quadruplex (GQ)-forming hexanucleotide repeat expansion (HRE) in the *C9orf72* (C9) gene has been found to be the most common cause of amyotrophic lateral sclerosis (ALS) and frontotemporal dementia (FTD) (collectively, C9ALS/FTD), implying the great significance of modulating C9-HRE GQ structures in C9ALS/FTD therapeutic treatment strategies. In this study, we investigated the GQ structures formed by varied lengths of C9-HRE DNA sequences d(GGGGCC)_4_ (C9-24mer) and d(GGGGCC)_8_ (C9-48mer), and found that the C9-24mer forms anti-parallel GQ (AP-GQ) in the presence of potassium ions, while the long C9-48mer bearing eight guanine tracts forms unstacked tandem GQ consisting of two C9-24mer unimolecular AP-GQs. Moreover, the natural small molecule Fangchinoline was screened out in order to be able to stabilize and alter the C9-HRE DNA to parallel GQ topology. Further study of the interaction of Fangchinoline with the C9-HRE RNA GQ unit r(GGGGCC)_4_ (C9-RNA) revealed that it can also recognize and improve the thermal stability of C9-HRE RNA GQ. Finally, use of AutoDock simulation results indicated that Fangchinoline binds to the groove regions of the parallel C9-HRE GQs. These findings pave the way for further studies of GQ structures formed by pathologically related long C9-HRE sequences, and also provide a natural small-molecule ligand that modulates the structure and stability of C9-HRE GQ, both in DNA and RNA levels. Altogether, this work may contribute to therapeutic approaches of C9ALS/FTD which take the upstream C9-HRE DNA region, as well as the toxic C9-HRE RNA, as targets.

## 1. Introduction

**Figure 1:**
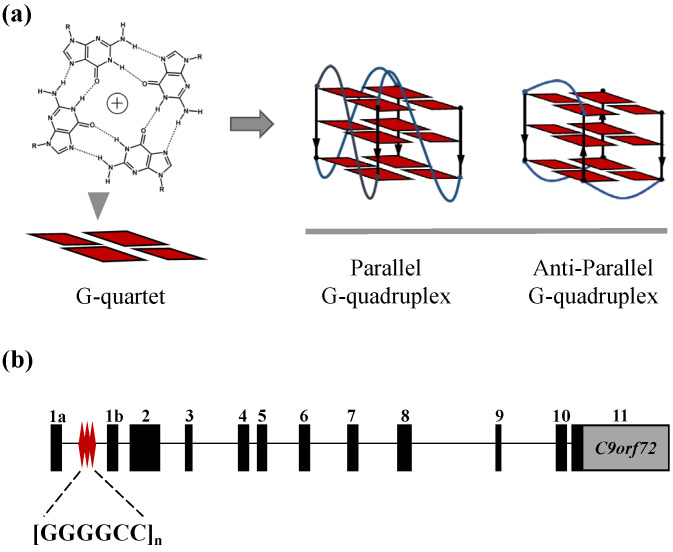
(**a**) Guanine rich oligonucleotides form G-quartets which stack on each other to form different topologies of G-quadruplexes. (**b**) Map of the genetic locus of HRE in the *C9orf72* gene.

Guanine-rich oligonucleotides are known to be able to form higher-order structures called G-quadruplexes (GQs) in the presence of monovalent cations [1,2,3] (Figure 1a). Numerous studies have shown that putative GQ-forming sequences are pervasively distributed across the human genome with enrichment in the regulatory elements of gene replication, transcription and translation, and thus implies the potent physiological role of GQs [4,5,6,7]. So far, GQs have been extensively studied as therapeutic targets for diseases treatment, especially for cancers [8,9,10,11,12,13]. In the past decade, however, there has been a growing exploration of tandem repeat GQs as their biological relevance to neurological disorders [14,15,16], such as fragile X syndrome (FXS), which is mainly caused by the GQ-forming trinucleotide repeat expansions in *FMR1* [17,18], and progressive myoclonus epilepsy (EPM1), which is reported to be related to the GQ-forming dodecamer repeat expansions in *CSTB* [19,20], has been discovered.

The C9ALS/FTD spectrum disorder represents two rare progressive neurodegenerative diseases. ALS is a fatal disease which is associated with the degeneration of motor neurons in the brain and spinal cord [21]. FTD is the second most common form of dementia in persons younger than 65 years of age, with characteristics of atrophy and neuronal loss in the frontal and temporal lobes of the brain [22]. ALS and FTD overlap extensively in their clinical presentation, pathology and, especially, genetics. They can both be caused by many mutations in the same set of genes, the most prevalent of which is a hexanucleotide repeat expansion (HRE) d(GGGGCC)_n_ in the chromosome 9 open reading frame 72 (*C9orf72*) gene [23,24] (Figure 1b). The d(GGGGCC)_n_ repeat is located in the promoter region of exon 1b in the *C9orf72* gene, and it has been reported that while the repeat number is under 24 (*n* ≤ 24) in most healthy individuals, it extends to hundreds and even thousands in C9ALS/FTD patients [25]. The detailed mechanism of how C9-HRE causes C9ALS/FTD is still unresolved, but previous studies have proposed that C9-HRE could form polymorphic secondary structures, including GQs in DNA and RNA levels which disturb the transcription and translation processes, leading to the loss of *C9orf72* protein, the accumulation of RNA foci and the expression of neurotoxic dipeptide-repeat proteins (DPRs) [26,27].

Due to the high pathological association of C9-HRE with C9ALS/FTD, genetic approaches targeting the repetitive region in DNA and RNA levels have been increasingly explored in the past several years. Stereopure antisense oligonucleotides were discovered to reduce RNA foci and the DPR gain-of-function toxicity in preclinical mice models [28,29]. A wide-spectrum GQ ligand, TMPyP4, was reported to be able to mitigate neurotoxicity in *Drosophila* models by altering the structure of C9-HRE RNA GQ, which causes an inhibition of the toxic repeat-associated non-AUG (RAN) translation [30,31,32]. Zhu [33] and other groups [34,35] have reported that small-molecule ligands targeting C9-HRE RNA GQ can significantly reduce RNA foci and DPRs levels, and thus ameliorate C9ALS/FTD pathology in vitro and in vivo. Although the pathological efficacy of small-molecule ligands targeting C9-HRE DNA GQs still needs to be addressed, it is posited that modulating the stability or topology of C9-HRE DNA GQ structures might contribute to the impediment of transcription within the C9-HRE locus, and thus reduce the overall C9-HRE-linked gain-of-function pathogenic mechanisms, such as ribonucleoprotein sequestration by RNA foci or the RAN translation of toxic DPRs [36]. All these studies make the regulation of C9-HRE GQ structures by small molecules a potent therapeutic strategy for the treatment of related C9ALS/FTD diseases.

Despite previous biophysical studies have examined the GQ structures adopted by varied lengths of C9-HRE DNA sequences with no more than five repeats [37,38,39,40,41], the structures of those bearing more than one tandem GQ motif that are highly associated with neurodegenerative diseases are still unclear, which hampers the strategy of screening small-molecule ligands to target the C9-HRE region. In this study, we investigated the GQ structures formed by both the short C9-HRE DNA sequence d(GGGGCC)_4_ (C9-24mer) that can form a single GQ, and the long d(GGGGCC)_8_ (C9-48mer) with two GQ motifs. Our results indicate that the C9-48mer forms an unstacked tandem structure consisting of two C9-24mer AP-GQs, suggesting that the GQ motifs may probably be arranged in a bead-on-a-string mode [42] in the long C9-HRE sequences. Moreover, we screened nine natural small molecules existing in traditional Chinese herbs and found that the bisbenzylisoquinoline alkaloid Fangchinoline can efficiently recognize and regulate both the C9-24mer single GQ and the C9-48mer unstacked tandem GQs to highly stable parallel topologies. We also evaluated the interaction of Fangchinoline with the toxic gain-of-function-related C9-HRE RNA GQ r(GGGGCC)_4_ (C9-RNA), and found that it can also recognize and significantly stabilize C9-RNA. Further docking results give an insight into the groove-binding mode of Fangchinoline with parallel C9-HRE GQs. Together, these findings expand our knowledge of the structures of DNA GQs formed by long C9-HRE sequences, which may serve as therapeutic targets to prevent C9ALS/FTD, and also provide a natural small-molecule ligand that can efficaciously modulate C9-HRE GQs both in DNA and RNA levels, which may have potential implications for altering C9ALS/FTD disease pathogenesis.

## 2. Results

### 2.1. The C9-HRE Long Sequence Forms Unstacked Tandem AP-GQ Structure in a Bead-on-a-String Mode

**Figure 2:**
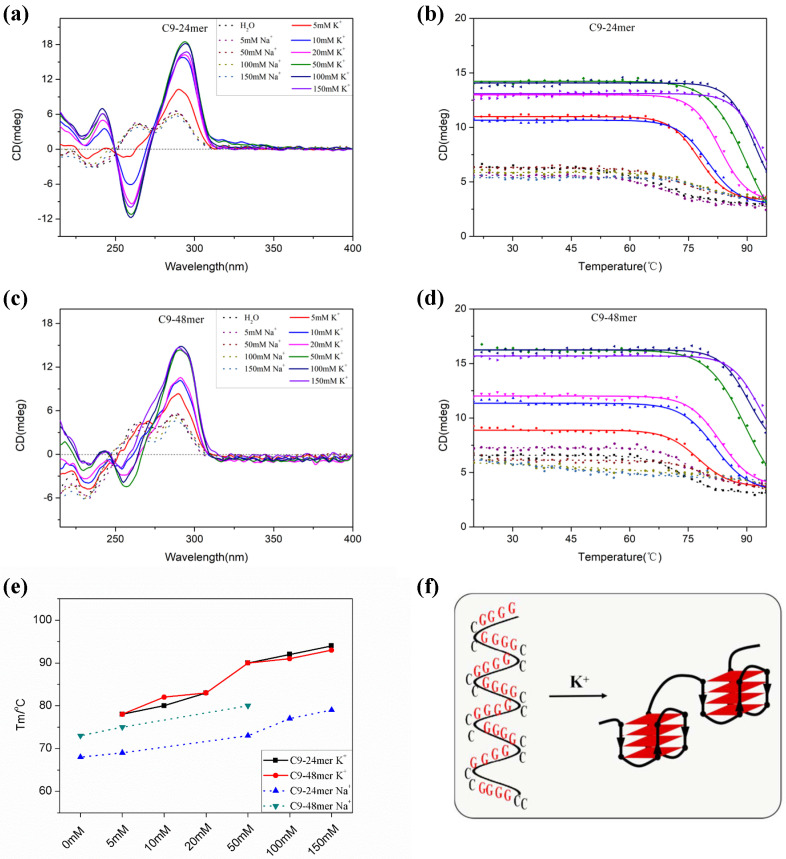
(**a**) The CD spectra and (**b**) CD variable temperature measurement recorded at 290 nm of 2 μM C9-24mer in a buffer containing 30 mM Tris-HCl (pH 7.4), 0–150 mM NaCl or KCl, as illustrated. (**c**) The CD spectra and (**d**) CD variable temperature measurement recorded at 290 nm of 1 μM C9-48mer in a buffer containing 30 mM Tris-HCl (pH 7.4), 0–150 mM NaCl or KCl, as illustrated. (**e**) The melting temperature (Tm) comparison between C9-24mer and C9-48mer under varied ionic conditions. (**f**) An illustration of C9-48mer forming a bead-on-a-string structure with each of the individual GQ units adopting AP-GQ conformation.

Previous NMR studies by Plavec’s group [37,38,39] have revealed that the short sequence C9-24mer, which represents the shortest model of C9-HRE able to form unimolecular GQ, mainly forms two major AP-GQ structures differing in the donor-acceptor directionalities of G-quartets in the presence of K^+^ ions. Here, we systematically investigated C9-24mer G-quadruplex formation under different monocation conditions through CD spectroscopy. As shown in Figure 2a, the CD absorption of C9-24mer in H_2_O and 5 to 150 mM Na^+^ solution are almost identical, with a negative absorption band around 236 nm, and two positive absorption bands around 263 nm and 288 nm, indicating the formation of mixed quadruplex structures containing GCGC tetrads, as the structure formation is insensitive to monovalent ionic conditions [43,44]. However, in the presence of 5 mM potassium ions, C9-24mer provides a CD spectrum with classic AP-GQ characteristics: a large maximum around 290 nm, a negative band around 260 nm and another small positive band around 240 nm [45]. Moreover, CD absorption increases significantly when the concentration of the potassium ions reaches 10 mM. Further increases of K^+^ concentration up to 150 mM give a slight absorption enhancement, indicating the strong K^+^ inducement of C9-24mer AP-GQ. It is notable that despite CD spectroscopy being a highly sensitive and reliable method to discriminate among various DNA secondary structures, it does not directly reflect strand orientation; therefore, the two AP-GQ topologies of C9-24mer in the presence of K^+^ ions resolved using the NMR method [38] cannot be distinguished by our CD experiment. To further evaluate the thermal stability of C9-24mer AP-GQ, we performed variable temperature measurement experiments. As shown in Figure 2b, the CD melting temperature of C9-24mer recorded at 290 nm in H_2_O or 5–150 mM Na^+^ solution (68 °C, 69 °C, 73 °C, 77 °C, 79 °C, respectively) is relatively lower than those in potassium solution, which is consistent with the CD absorption result that C9-24mer may form mixed structures consisting of GGGG and GCGC tetrads. However, in the presence of 5–150 mM K^+^ solution, the C9-24mer topology is much more thermally stable, with the CD melting temperature increasing gradually from 78 °C, 80 °C, 83 °C, 90 °C, 92 °C, to 94 °C, respectively, suggesting that C9-24mer can form a stable AP-GQ structure under near-physiological ionic conditions.

Next, using the C9-48mer oligonucleotide which contains eight guanine tracts that can form two GQ motifs as a model, we evaluated the higher-order structure formation of C9-HRE long sequences. As shown in Figure 2c, the CD absorption of C9-48mer in H_2_O, 5–150 mM of Na^+^ solution or K^+^ solution follows almost the same pattern as that of C9-24mer, demonstrating that the potassium ions induced AP-GQ formation by the C9-HRE long sequences, yet it is still not clear how the individual G-tracts are arranged in the AP-GQ of the long C9-HRE sequences. It has been suggested that the long C9-HRE sequences may form a bead-on-a-string structure with each of the individual GQ motifs adopting an AP-GQ conformation [14]; it has also been proposed that the long C9-HRE sequence d(GGGGCC)_8_ may adopt a quadruplex structure consisting of GGGG tetrads sandwiched between GCGC tetrads in the presence of Na^+^ or Li^+^ solutions [43]. Here, through the use of CD variable temperature measurement, we systematically investigate the thermal stability of C9-48mer and compare it to that of C9-24mer to evaluate the possible conformation of long C9-HRE sequences under different monovalent cation conditions. As shown in Figure 2d, the thermal stability of C9-48mer is enhanced as the concentration of the monovalent cation Na^+^ or K^+^ increases. A further comparison of the Tm values between the short C9-24mer and long C9-48mer under variable monovalent cation conditions (Figure 2e, dot lines) shows that the thermal stability of C9-48mer (73 °C, 75 °C, 80 °C, respectively) is significantly higher than that of C9-24mer under 0–50 mM Na^+^ conditions. This could be explained by, and is consistent with, CD absorption results that C9-HRE sequences form a mixed quadruplex structure with GGGG tetrads sandwiched between GCGC tetrads under H_2_O or Na^+^ conditions. Since the longer C9-48mer consists of twice as many stacked tetrads as C9-24mer, it exhibits a much higher thermal stability than C9-24mer. In fact, under 100 mM or 150 mM Na^+^ conditions, the melting curve of C9-48mer does not fit a two-state transition model anymore, and the Tm value cannot be determined, indicating the existence of intermediate topology states under these conditions. However, in the presence of 5–150 mM K^+^ solution, the melting temperature of C9-48mer (78 °C, 82 °C, 83 °C, 90 °C, 91 °C, 93 °C, respectively) is almost identical to that of C9-24mer (Figure 2e, solid lines), suggesting that the individual GQ units in C9-48mer are composed of four repeats and adopt an AP-GQ conformation just like the C9-24mer does, while the unstacked tandem individual units are connected by a CC linker, as illustrated in Figure 2f. Together, our results indicate that the long C9-HRE sequence exhibits structure polymorphism under water or sodium conditions, while the individual GQ units adopt AP-GQ conformation and are arranged in a bead-on-a-string mode, rather than a stacked structure, under physiological potassium conditions.

### 2.2. Fangchinoline Alters the C9-HRE Topology to Stable Parallel GQ

Since the C9-HRE sequences forms a bead-on-a-string AP-GQ structure, it is reasonable to use the C9-24mer consisting of a single AP-GQ unit as a model to screen ligands that can interact with the C9-HRE GQs. Classical GQ-binding ligands have either a π-conjugated structure for stacking on the G-tetrad, or a flexible structure for binding with the loop or groove region. Hence, we selected nine natural small molecules (Figure S1) that mainly exist in traditional Chinese herbs, eight of which (Tanshinone I, Tanshinone IIA, Dihydrotanshinone I, Psoralen, Isopsoralen, α-Mangostin, Alkannin and Magnolol) have conjugated structures and one of which is a flexible bisbenzylisoquinoline alkaloid (Fangchinoline), and evaluated their interaction with C9-24mer AP-GQ through CD experiments. First, four equivalents of each molecule were added to the C9-24mer ODN in 5 mM K^+^ solution and pre-annealed overnight; the recorded CD spectra show that the π-conjugated small molecules had little effect on the formation of C9-24mer AP-GQ, while the bisbenzylisoquinoline Fangchinoline effectively altered CD absorption from 290 nm to 265 nm (Figure S2a), which is a characteristic of parallel GQ (P-GQ). Further CD melting experiments supported this result, as the π-conjugated small molecules hardly impacted the C9-24mer thermal stability, while Fangchinoline significantly enhanced the Tm value by 6 °C (Figure S2b).

**Figure 3:**
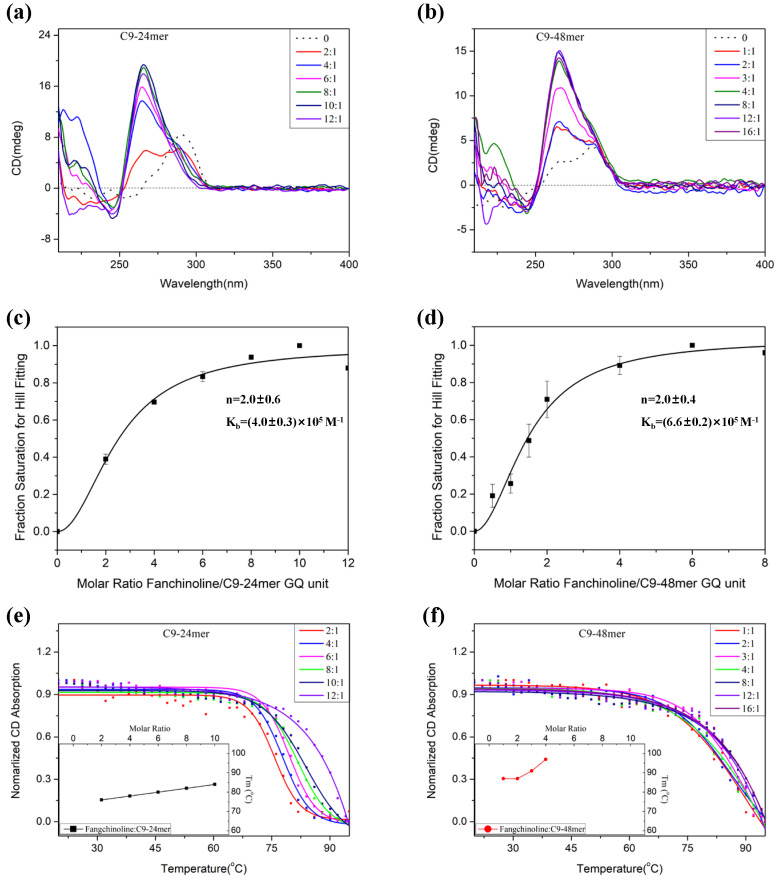
The CD spectra of (**a**) 2 μM C9-24mer and (**b**) 1 μM C9-48mer with varied equivalents of Fangchinoline in 5 mM KCl, 30 mM Tris-HCl (pH 7.4) solution. The curves of the Hill fitting for the binding constant and stoichiometry calculations of Fangchinoline with (**c**) C9-24mer and (**d**) C9-48mer GQ. The data represent an average of two replicates. The CD melting curve and melting temperature Tm (inner panel) of (**e**) C9-24mer and (**f**) C9-48mer recorded at 265 nm with varied equivalents of Fangchinoline.

Next, we systematically investigated the interactions between Fangchinoline and both the short C9-24mer and the long C9-48mer sequences. As shown in Figure 3a, when two equivalents of Fangchinoline were added to the solution of C9-24mer in 5 mM K^+^ condition, the positive peak at 290 nm decreased and a new peak at 265 nm appeared in the CD spectrum. As the molar ratio of Fangchinoline increased to four, six and eight, the absorption at 290 nm decreased and that at 265 nm was gradually enhanced. At a molar ratio of 10, the CD spectrum revealed a single strong positive peak at 265 nm, indicating that C9-24mer was induced to pure P-GQ conformation under this condition. The same method was applied to evaluate the interaction of Fangchinoline with the long C9-48mer sequence which represents the bead-on-a-string C9-HRE structure. Similar to those of C9-24mer, the CD spectra of C9-48mer with variable equivalents of Fangchinoline also revealed a conformation transition from AP-GQ to P-GQ (Figure 3b), as the characteristic AP-GQ absorption at 290 nm disappeared and the P-GQ absorption at 265 nm was gradually enhanced as the molar ratio of Fangchinoline:C9-48mer increased. To calculate the binding constant and stoichiometric ratio of Fangchinoline with C9-HRE GQ, the curve of a normalized CD absorption at 265 nm plotted to the molar ratio of Fangchinoline/C9-HRE GQ units was fitted with the Hill equation. As shown in Figure 3c,d, while Fangchinoline binds to the GQ units in C9-24mer and C9-48mer with a similar stoichiometry of approximately 2.0, which is consistent with previously reported results that Fangchinoline binds to other GQs, such as *bcl-2* and *c-kit*, with a stoichiometry of two [46,47], its binding constant with C9-48mer ((6.6 ± 0.2) × 10^5^ M^−1^) is much higher than that with C9-24mer ((4.0 ± 0.3) × 10^5^ M^−1^), indicating the more efficient interaction of Fangchinoline with the two-bead-on-a-string C9-48mer GQ than that with the individual C9-24mer GQ. We also investigated the selectivity of Fangchinoline binding between GQ and duplex DNA structures through CD and PAGE experiments, and found that it exhibits no binding affinity to the randomly generated duplex (Dup I + II), while it is able to induce C9-HRE P-GQ formation from the polymorphic secondary structures adopted by a mixture of d(GGGGCC)_4_ and its complementary C-rich strand d(GGCCCC)_4_ (Figure S3).

Furthermore, the thermal stability of C9-HRE GQ in the presence of varied equivalents of Fangchinoline was evaluated. Not surprisingly, the Tm value of C9-24mer GQ recorded at 265 nm increased gradually to 84 °C as the molar ratio of Fangchinoline increased to 10, and was out of detectable range at a molar ratio of 12 (Figure 3e, inner panel), suggesting that Fangchinoline can effectively alter the C9-24mer topology to a stable P-GQ. Moreover, the melting curves of Fangchinoline with C9-48mer recorded at 265 nm reveal a much higher thermal stability than that of C9-24mer. In fact, the calculated Tm value reached 90 °C even at a Fangchinoline:C9-48mer molar ratio of one, and was out of the detectable range when the molar ratio was higher than four (Figure 3f, inner panel). Therefore, it can be inferred that Fangchinoline may probably induce the C9-HRE long sequences to form stacked tandem P-GQ structures, which can explain the extremely high thermal stability of C9-48mer in the presence of Fangchinoline.

**Figure 4:**
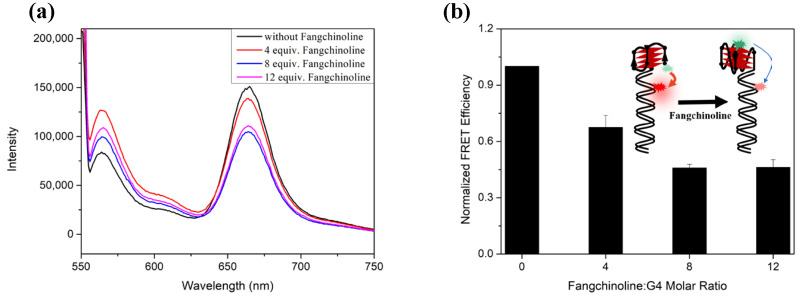
(**a**) The fluorescence emission spectra excited at 551 nm and (**b**) the normalized FRET efficiency of 2 μM FRET I and II each, with 0, 8, 16 or 24 μM Fangchinoline in 100 mM KCl, 30 mM Tris-HCl, pH 7.4. The inner panel in (**b**) illustrates the FRET efficiency decrease caused by the Fangchinoline-induced C9-24mer GQ conformation transition.

We also performed FRET experiments to confirm this result. The previously reported FRET system developed by Balasubramanine and others [48,49], which can sensitively distinguish between AP-GQ and P-GQ conformations, was applied to the C9-24mer sequence. We first evaluated whether the C9-24mer repeats attached with a 35 nt overhang duplex (FRET I + II) could form AP-GQ in 100 mM K^+^ solution using CD spectroscopy. As shown in Figure S4, the CD absorption of FRET I + II exhibits a broad positive peak around 290 nm, which indicates a combination structure of AP-GQ and duplex. As Fangchinoline was added to the solution, the broad positive peak shifteds to 265 nm, indicating the topology of the C9-24mer sequence in this system was altered to P-GQ, just as the free C9-24mer oligo was. Next, with the excitation wavelength set at 551 nm, the fluorescence emission spectra of I + II with varied equivalents of Fangchinoline were recorded. As shown in Figure 4a, the fluorescence intensity of the Cy5 emission at 670 nm decreased gradually as the molar ratio of Fangchinoline: (I + II) increased from 0 to 12, and the normalized FRET efficiency decreased to 0.67, 0.46 and 0.46, respectively (Figure 4b). This demonstrates that C9-24mer was induced to P-GQ by Fangchinoline, and thus caused an increase in the distance between the two dyes, Cy3 and Cy5, which gives a lower FRET efficiency, as illustrated in the Figure 4b inner panel.

### 2.3. Fangchinoline Can Recognize and Stabilize C9-HRE RNA GQ

**Figure 5:**
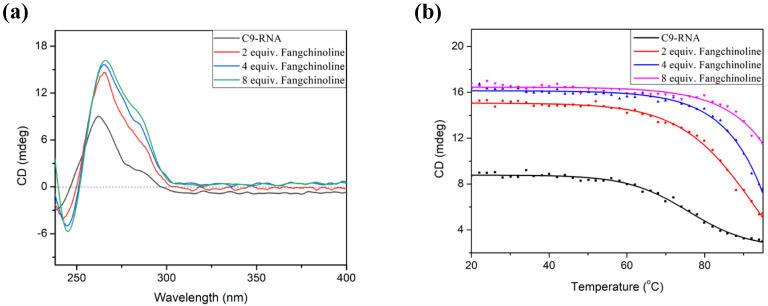
(**a**) The CD spectra and (**b**) CD melting curve of 5 μM C9-RNA with varied equivalents of Fangchinoline in 5 mM KCl, 30 mM Tris-HCl (pH 7.4) solution.

The abortive transcription of *C9orf72* generates G/C-rich RNA, including r(GGGGCC)_n_, that can form RNA GQ. Small-molecule ligands interacting with C9-HRE RNA GQ have been reported to be able to ameliorate the *C9orf72* gain-of-function related to C9ALS/FTD pathology [33,34]. Therefore, we further evaluated the interaction of Fangchinoline with the C9-HRE RNA GQ formed by r(GGGGCC)_4_ (C9-RNA). As shown in Figure 5a, C9-RNA forms parallel GQ in K^+^ solution, which is demonstrated by the positive CD absorption at 262 nm. When two to eight equivalents of Fangchinoline were added and co-annealed with C9-RNA, the positive absorption around 262 nm was enhanced drastically from 9.0 to 16.2 mdeg, indicating that Fangchinoline could recognize and induce parallel C9-RNA GQ formation. Moreover, the CD melting temperature of the C9-RNA GQ increased 17 °C in the presence of two equivalents of Fangchinoline (76 vs. 93 °C) and was out of detectable range in the presence of four or eight equivalents of Fangchinoline. Altogether, these results indicate that the C9-HRE DNA GQ ligand Fangchinoline can also bind to and stabilize HRE RNA GQ.

### 2.4. Fangchinoline Is a Groove-Binder to the Parallel C9-HRE GQ

**Figure 6:**
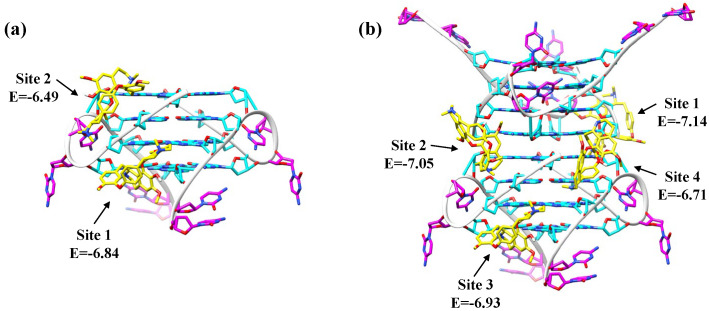
Molecular docking analysis showing the best docking poses of Fangchinoline with (**a**) the C9-HRE P-GQ unit and (**b**) the stacked dimeric C9-HRE P-GQs taken from the Protein Data Bank (PDBID: 7ECH). The G and C residues are colored cyan and magenta, respectively. The Fangchinoline is colored yellow. The hetero atoms O, N and P are colored red, blue and orange, respectively. The binding free energies are labeled right below each site with the unit of kcal/mol.

To get an idea of whether Fangchinoline could bind directly to C9-HRE AP-GQ and alter it into P-GQ, or if it binds to the unfolded C9-HRE and induces the formation of P-GQ simultaneously, we added Fangchinoline to the pre-generated C9-HRE AP-GQ solution without further annealing, and found no induced CD absorption around 260 nm (Figure S5), indicating that Fangchinoline should be a P-GQ rather than an AP-GQ binder. Based on this, we performed an in silico docking analysis using Autodock4.2 [50] to gain insight into the binding mode of Fangchinoline with the parallel C9-HRE GQ. Since an accurate NMR or crystal structure of the C9-HRE P-GQ formed by d(GGGGCC)_4_ is not available yet, the biological assembly structure of d(GGGGGCC)_2_ in K^+^ solution (PDBID: 7ECH) [41] resolved by crystallography was used to obtain the single C9-HRE P-GQ unit, which corresponds to C9-24mer GQ, and the stacked units, which corresponds to C9-48mer GQ. As the previously calculated stoichiometry of Fangchinoline with the C9-HRE GQ unit was approximately equal to two (Figure 3c,d), the docking conformations in the clusters with the lowest two or four binding energies were selected to show the most probable binding sites of C9-24mer or C9-48mer. As shown in Figure 6a, according to the docking results, Fangchinoline prefers to bind to the two-groove regions formed between two GGGG strands (Site 1) or a GGGG strand and a CC loop (Site 2) of the C9-HRE P-GQ unit; it binds in a similar pattern to the stacked dimeric C9-HRE P-GQs (Figure 6b), with two groove-binding sites formed between a GGGG strand and a CC loop (Site 1 and Site 2), and another two sites between adjacent GGGG strands (Site 3 and Site 4). Moreover, the module of the calculated binding energy of Fangchinoline with the C9-HRE P-GQ unit (−6.67 kcal/mol on average for Site 1 and Site 2) was lower than that with the dimeric P-GQs (−6.96 kcal/mol on average for Site 1 to Site 4), which is consistent with the previous binding constant result calculated by Hill fitting using the CD method, and may be caused by extra non-electrostatic interactions of Fangchinoline with the interface groove regions in the dimeric P-GQs (Figure 6b, Site 1 and Site 2). However, it should be noticed that the conformational flexibility is not modeled in the *in silico* docking process, and thus the precise interaction and the complex conformation still need further exploration using either molecular dynamics (MD) simulations, as described by Gil’s and Bhowmik’s groups [51,52,53,54], or NMR and crystallography studies.

## 3. Discussion

C9-HRE is highly pathologically, being associated with C9ALS/FTD, and is considered to be a potent therapeutic target for related diseases treatment. Although the precise mechanism by which C9-HRE causes C9ALS/FTD is still unclear, the structure evaluation of long C9-HRE sequence and its conformation regulation in DNA as well as RNA level provides important potential for the C9-HRE related C9orf72 transcription and subsequential RNA foci and DPRs formation regulation. Previous studies have mainly focused on the recognition and regulation of r(GGGGCC)_n_ GQ structures, which are reported to be able to recruit RNA binding proteins (RBPs) to form toxic RNA foci and related to DPRs accumulation, while studies on the structure and regulation of HRE DNA GQ are relatively insufficient. It has been reported that C9-HRE DNA is highly associated with the binding of trimethylated histones, including H3K9me3, H3K27me3 and H3K79me3, which further inhibits the transcription of *C9orf72* [55]. *C9orf72*‘s most abundant transcript variant, V2, was reported to be reduced around 50%, and C9ORF72 protein levels are correspondingly reduced in C9ALS/FTD patients [56]. A recent study by De Vos’ group indicated that C9ORF72 haploinsufficiency is the cause of synaptic dysfunction in C9ALS/FTD [57]. Moreover, the C9-HRE GQ DNA locus on the promoter region of *C9orf72* V2 on the sense strand, on which GQ structure stabilization was reported to be able to facilitate the transcription of the related gene [58]. Conformation regulation and stabilization of the DNA GQ may disturb the interaction of C9-HRE with trimethylated histones and facilitate transcription, and thus reduce *C9orf72* loss-of-function. On the contrary, DNA GQ formation and structure stabilization may inhibit C9-HRE transcription of the anti-sense strand, which may contribute to the reduction of C9-HRE-linked gain-of-function, such as RNA foci and toxic DPRs accumulation [59].

Herein, our study reveals that the C9-HRE sequence d(GGGGCC)_8_ forms an AP-GQ structure composed of two unstacked individual d(GGGGCC)_4_ AP-GQ units, implying that the long C9-HRE sequence containing more than one GQ unit may probably form a bead-on-a-string structure, just like human telomeric DNAs [42]. Moreover, the bisbenzylisoquinoline alkaloid Fangchinoline, which was previously reported to be able to bind with three-layered GQs [46,47], can also recognize and induce the four guanines-containing G-tracts GGGGCC to form a stable parallel topology, which may stack on each other, forming the super-stable higher-order structure of the long C9-HRE sequence. Furthermore, Fangchinoline is also able to bind and stabilize the parallel C9-HRE RNA GQ. An in silico docking analysis revealed that Fangchinoline is a groove binder to the parallel C9-HRE GQ. Together, these results could aid in further underlying mechanism studies of C9-HRE-related C9ALS/FTD and pathological regulation in both DNA and RNA levels related to C9-HRE loss-of-function and gain-of-function toxicity.

## 4. Materials and Methods

### 4.1. Materials

The oligonucleotides used in this study are listed in Table S1. All the DNA oligos were synthesized by Sangon Biotech (Shanghai, China), and the RNA oligos were synthesized by Genscript Biotech (Nanjing, China). All the oligos were dissolved in Milli-Q water and quantified using a Thermo Scientific Nanodrop 2000 spectrophotometer (Thermo Fisher Scientific, Waltham, MA, USA) to generate a stock solution at 100 μM. Natural small molecules (analytical grade) were purchased from Yuanye Biotech (Shanghai, China). Tanshinone I, Tanshinone IIA and Dihydrotanshinone I were dissolved in DMSO; all the other small molecules were dissolved in pure methanol to a stock concentration of 1 mM.

### 4.2. Circular Dichroism (CD) Spectroscopy

CD experiments were performed using a J-815 CD spectrometer (JASCO, Tokyo, Japan) to evaluate the topologies of the C9-HRE GQs. The DNA oligos were diluted to 2 μM of GQ motifs (2 μM for C9-24mer and 1 μM for C9-48mer), and the RNA oligos were diluted to 5 μM of GQ motifs in 30 mM Tris-HCl (pH 7.4) buffer containing different concentrations of monovalent cations as described. For the complex samples of DNA or RNA with the small molecules, the CD absorption of the corresponding pure small molecule solution was recorded and subtracted as background. All the samples were pre-annealed at 90 °C for 10 min and then slowly cooled down to 25 °C (more than 8 h) before being tested in a 1.0 cm (for the DNA samples) or 0.1 cm (for the RNA samples) path-length cuvette, with a scanning wavelength range from 400 to 220 nm at 1 nm intervals. Three scans were averaged for each spectrum. The Hill equation (y = Vmax × x*^n^*/(k*^n^* + x*^n^*)) was fit to the data to calculate the binding constant and the number of binding sites of Fangchinoline with the C9-24mer or C9-48mer GQ units.

### 4.3. CD Melting Experiment

CD melting experiments were carried out using a Peltier temperature control unit equipped in the J-815 CD spectrometer (JASCO). The samples were tested in a 1.0 cm (for the DNA samples) or 0.1 cm (for the RNA samples) cuvette, put in the cell holder and heated from 20 °C to 95 °C, with a temperature gradient of 2 °C. The heating rate was set at 2 °C/min and measurements were triggered when each target temperature was reached and kept within ±0.10 °C for at least 5 s to avoid any substantial hysteresis.

### 4.4. Polyacrylamide Gel Electrophoresis (PAGE) Experiment

PAGE experiments were performed to evaluate the binding selectivity of Fangchinoline towards the C9-HRE GQ over duplex DNA structures. An amount of 2 μM of Dup I and II each were mixed with either 8 μM or without Fangchinoline in a 5 mM KCl, 30 mM Tris-HCl (pH 7.4) buffer, pre-annealed at 90 °C for 10 min and then slowly cooled down to 25 °C to generate the random duplex DNA sample with or without Fangchinoline, respectively. The mixture of the C9-24mer G-strand with an equal molar of its complementary C-strand (C9-24mer-C) was generated in the same way as above. A non-denaturing PAGE was performed on a 15% polyacrylamide (Acrylamide/Bis 19:1) gel in a 1 × TBE buffer containing 90 mM Tris-Borate and 2 mM EDTA (pH = 8.0). Electrophoresis was run at 90 V for 2.5 h and imaged using a BioRad Gel Doc XR+ system (Bio-Rad Laboratories, Hercules, CA, USA).

### 4.5. Fluorescence Resonance Energy Transfer (FRET) Experiment

FRET experiments were performed using the FLS980 Spectrometer (Edinburgh Instruments, Livingston, UK) to evaluate the C9-HRE GQ conformation transition. As previously reported [48,49], the C9-HRE GQ unit was attached to a 35 nt overhang and labeled with Cy3 fluorophore at the 5′ terminus (Table S1, FRET I); the complementary sequence of the 35 nt overhang was labeled with the fluorophore Cy5 at the eighth thymine from the 3′ terminus (Table S1, FRET II). The fluorophore-coupled oligos were mixed equally in a 100 mM KCl, 30 mM Tris-HCl (pH 7.4) solution to a final concentration of 2 μM with 0, 4, 8 or 12 equivalents of Fangchinoline, as described. The excitation/emission wavelength for the Cy3 and Cy5 dyes were optimized to be 551/565 nm and 648/670 nm, respectively. Thus, all the samples were excited at 551 nm and an emission peak area ratio of 670/565 nm was used for the FRET efficiency calculation.

### 4.6. AutoDock Simulation

The parallel C9-HRE GQ structures containing a single and two GQ units were taken from the crystal structure of d(GGGGGCC)_2_-K Biological Assembly 1 (PDBID: 7ECH) [41] and set as the docked input macromolecules for AutoDock analysis using AutoDockTools (version 1.5.6) [50]. The chemical structure of the small molecule Fangchinoline was constructed using ChemDraw (version 18.0.0). The Gasteiger partial charges were assigned to the atoms of the macromolecules and Fangchinoline. The Lamarckian genetic algorithm (LGA) embedded in ADT4.2 was adopted to perform molecular docking studies of 100 GA runs, with each of the populations containing 150 individuals and a maximum of 2.5 × 10^6^ energy evaluations. The final docked conformations were clustered, ranked by their binding energy, and the docking poses corresponding to the lowest binding energies were selected as the probable binding sites. Molecular graphics were generated using the Chimera (version 1.13.1) package [60].

## Data Availability

Not applicable.

## Supplementary Materials

The following supporting information can be downloaded at: https://www.mdpi.com/article/10.3390/molecules28124671/s1, Figure S1: Chemical structure of the natural small molecules used in this study; Figure S2: (a) CD spectra and (b) the melting temperature difference (ΔTm) of 2 μM C9-24mer in the presence of 8 μM of different natural small molecules. Conditions: 5 mM KCl, 30 mM Tris-HCl, pH 7.4.; Figure S3: (a) The CD spectrum of Dup I + II (black dashed line) exhibits a broad positive band around 280 nm which is a characteristic of duplex DNA. The alkaloid Fangchinoline does not affect the absorption of the duplex (black solid line), indicating no interaction between Fangchinoline and the duplex DNA. The CD spectrum of C9-24mer G + C (red dashed line) shows two positive peaks around 288 and 263 nm, suggesting the existence of polymorphic topologies, such as hairpin and duplex structures. Fangchinoline induces a strong absorption band around 266 nm (red solid line), indicating that P-GQ forms under this condition. (b) The electrophoresis bands of Dup I + II and C9-24mer G + C with or without Fangchinoline. The band of C9-24mer G + C in the presence of Fangchinoline (G + C + Fang.) clearly shows the induced GQ formation.; Figure S4: The CD spectra of 2 μM sequence I and II each, with 0, 4, 8, 12 or 16 μM of Fangchinoline in 100 mM KCl, 30 mM Tris-HCl, pH 7.4; Figure S5: The CD spectra of pre-generated 2 μM C9-24mer AP-GQ (red dashed line) or 1 μM C9-48mer AP-GQ (blue dashed line) by pre-annealing at 90 °C for 10 min and then slowly cooled down to 25 °C overnight. Incubation of 8 μM of Fangchinoline with the pre-generated AP-GQs at 37 °C for 5 h rarely affects the absorption bands (black and blue solid lines). Conditions: 5 mM KCl, 30 mM Tris-HCl, pH 7.4.; Table S1: The oligonucleotides used in this study.
