# Supplementary material for: G-Quadruplexes Formation by the C9orf72 Nucleotide Repeat Expansion d(GGGGCC)n and Conformation Regulation by Fangchinoline
Source: Molecules. 2023 Jun 9;28(12):4671. doi: 10.3390/molecules28124671 (PMC10300829; doi:10.3390/molecules28124671)
Supplement: Supplementary file 1 [file molecules-28-04671-s001.zip › molecules-2393182-supplementary.pdf]

# G-Quadruplexes Formation by the *C9orf72* Nucleotide Repeat Expansion d(GGGGCC)$_n$ and Conformation Regulation by Fangchinoline

**Yun Zhang [1,†], Junliu Huang [1,†], Kainan Yu [1] and Xiaojie Cui [1,2,*]**

[1] College of Life and Environmental Sciences, Minzu University of China, Beijing 100081, China
[2] Key Laboratory of Ecology and Environment in Minority Areas (Minzu University of China), National Ethnic Affairs Commission, Beijing 100081, China
* Correspondence: xiaojiecui@muc.edu.cn; Tel.: +86-13811634731
† These authors contributed equally to this work.

Table S1. The oligonucleotides used in this study.

| Code | Sequence (5'-3') |
|---|---|
| C9-24mer | GGGGCCGGGGCCGGGGCCGGGGCC |
| C9-48mer | GGGGCCGGGGCCGGGGCCGGGGCCGGGGCCGGGGCCGGG GCCGGGGCC |
| C9-RNA | r(GGGGCCGGGGCCGGGGCCGGGGCC) |
| FRET I | Cy3- <u>GGGGCCGGGGCCGGGGCCGGGGCC</u>AGAGGTAAAAGGATAA TGGCCACGGTGCGGACGGC |
| FRET II | GCCGTCCGCACCGTGGCCATTATCCTT-T/Cy5-TACCTCT |
| Dup I | GGGCATAGTGCGTGGCGTTTAGC |
| Dup II | GCTAAACGCCACGCACTATGCCC |
| C9-24mer-C | GGCCCCGGCCCCGGCCCCGGCCCC |

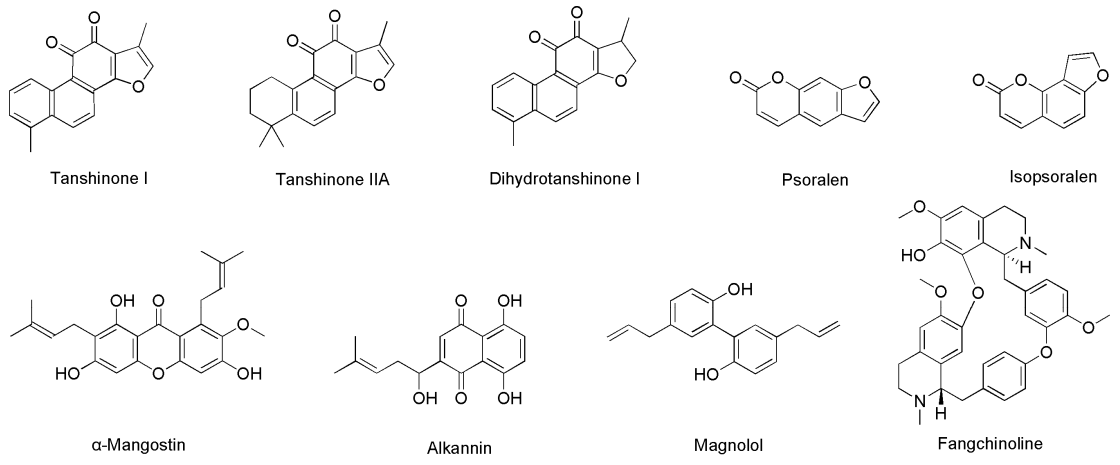


Figure S1. Chemical structure of the natural small molecules used in this study.

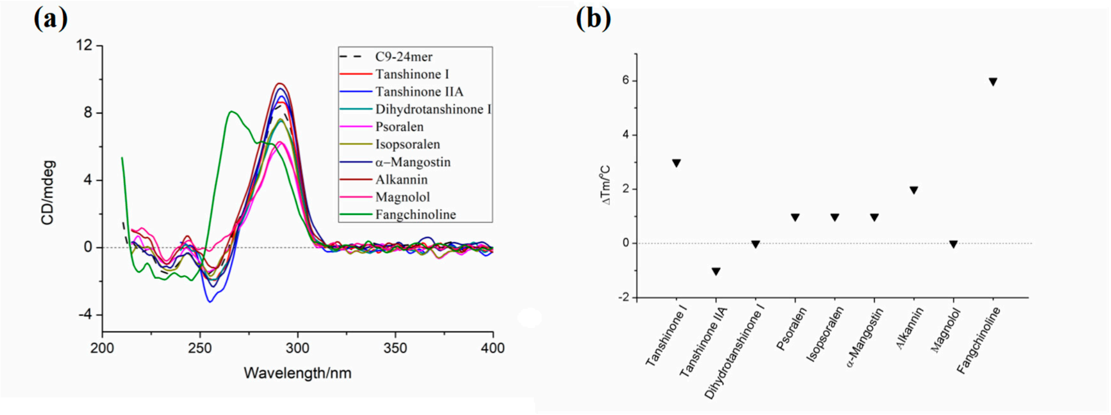


**Figure S2**. (a) CD spectra and (b) the melting temperature difference ($\Delta T_m$) of 2μM C9-24mer in the presence of 8μM of different natural small molecules. Conditions: 5mM KCl, 30mM Tris-HCl, pH 7.4.

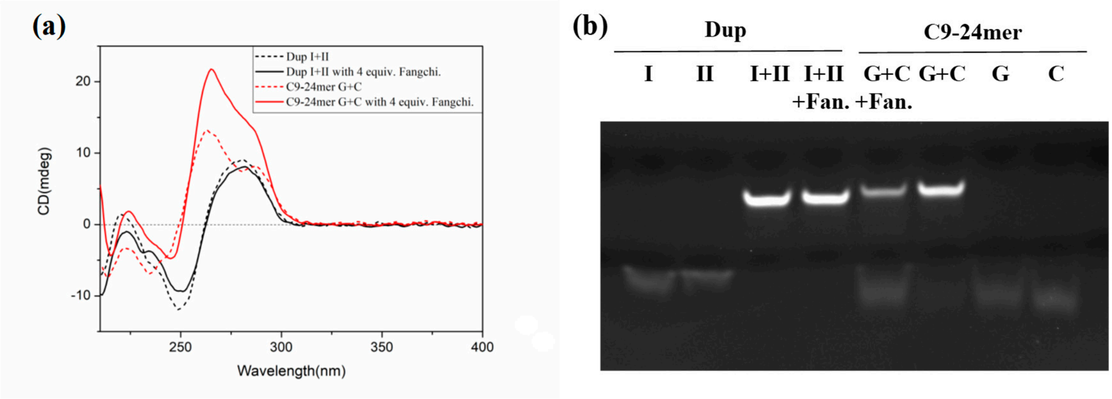


**Figure S3**. (a) The CD spectrum of Dup I+II (black dashed line) exhibits a broad positive band around 280 nm which is a characteristic of duplex DNA. The alkaloid Fangchinoline doesn't affect the absorption of the duplex (black solid line), indicating no interaction between Fangchinoline and the duplex DNA. The CD spectrum of C9-24mer G+C (red dashed line) shows two positive peaks around 288 and 263 nm, suggesting the existence of polymorphic topologies like hairpin and duplex structures. Fangchinoline induces a strong absorption band around 266 nm (red solid line), indicating P-GQ forms under this condition. (b) The electrophoresis bands of Dup I+II and C9-24mer G+C with or without Fangchinoline. The band of C9-24mer G+C in the presence of Fangchinoline (G+C+Fang.) clearly shows the induced GQ formation.

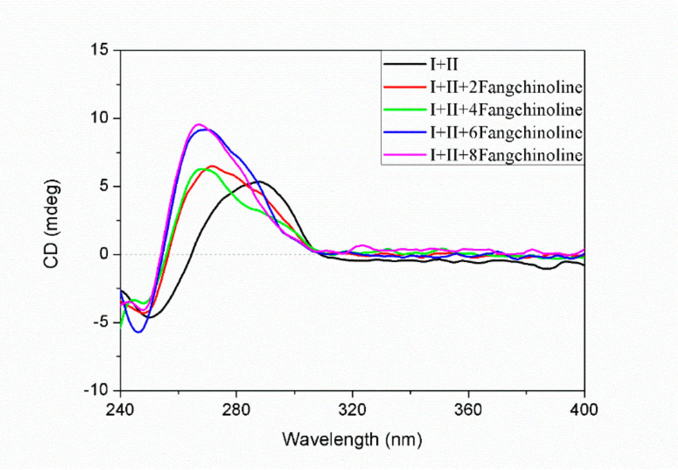


**Figure S4**. The CD spectra of 2μM sequence I and II each, with 0, 4, 8, 12, 16 μM of Fangchinoline in 100mM KCl, 30mM Tris-HCl, pH 7.4.

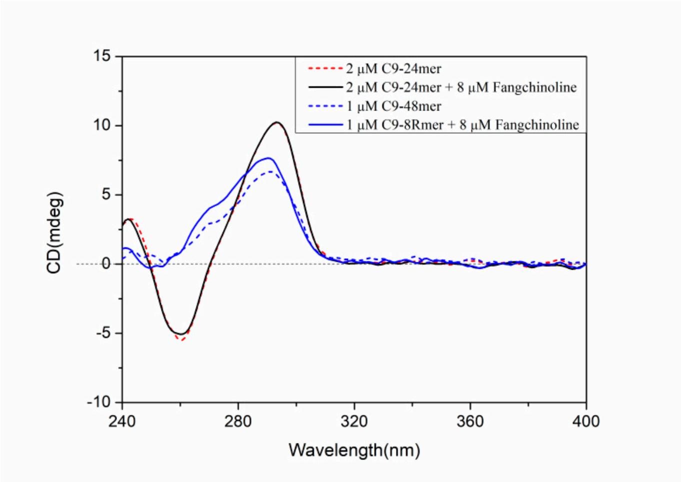


**Figure S5**. The CD spectra of pre-generated 2 μM C9-24mer AP-GQ (Red dashed line) or 1 μM C9-48mer AP-GQ (Blue dashed line) by pre-annealing at 90°C for 10 min and then slowly cooled down to 25°C overnight. Incubation of 8 μM of Fangchinoline with the pre-generated AP-GQs at 37°C for 5 h rarely affects the absorption bands (Black and blue solid lines). Conditions: 5mM KCl, 30mM Tris-HCl, pH 7.4.
